# Bridging Muscle and Bone Health: Rectus Femoris Ultrasound Parameters Predict Osteoporosis and Identify Low Muscle Mass in Romanian Postmenopausal Women

**DOI:** 10.3390/jcm14186531

**Published:** 2025-09-17

**Authors:** Miruna M. Soare, Andrea I. Gasparik, Horatiu V. Popoviciu, Ionela M. Pascanu

**Affiliations:** 1 Doctoral School of Medicine and Pharmacy, George Emil Palade University of Medicine, Pharmacy, Science, and Technology of Targu Mures, 540142 Targu Mures, Romania; 2 Department of Public Health and Health Management, George Emil Palade University of Medicine, Pharmacy, Science, and Technology of Targu Mures, 540142 Targu Mures, Romania; andrea.gasparik@umfst.ro; 3 Department of Rheumatology, George Emil Palade University of Medicine, Pharmacy, Science, and Technology of Targu Mures, 540142 Targu Mures, Romania; horatiu.popoviciu@umfst.ro; 4 Department of Endocrinology, George Emil Palade University of Medicine, Pharmacy, Science, and Technology of Targu Mures, 540142 Targu Mures, Romania; ionela.pascanu@umfst.ro

**Keywords:** sarcopenia, osteoporosis, muscle mass, rectus femoris, ultrasonography

## Abstract

**Background/Objectives**: Sarcopenia is characterized by a decline in muscle mass and function. Its association with osteoporosis—referred to as osteosarcopenia—is linked to increased risks of falls, fractures, frailty, and mortality. Therefore, there is a growing need for accurate and accessible tools to assess muscle mass. Ultrasonography has emerged as a promising modality in recent years. The aim of our study was to compare rectus femoris ultrasound parameters in postmenopausal women with osteoporosis to healthy controls and to evaluate its diagnostic performance against a reference method. **Materials and Methods**: A cross-sectional prospective study was conducted including 88 postmenopausal women with a mean age of 65.7 ± 7.5 years. Functional status was evaluated using handgrip strength and gait speed. Rectus femoris ultrasonography was performed, measuring muscle thickness (MT), cross-sectional area (CSA), pennation angle (PA), and echo intensity (EI). Body composition was analyzed using bioelectrical impedance analysis, and appendicular skeletal muscle mass (ASM) was estimated using a validated predictive equation. All participants had undergone dual-energy X-ray absorptiometry within the previous year, and FRAX scores were calculated. **Results**: Women with osteoporosis had significantly lower muscle thickness compared to controls after adjusting for age and BMI. Rectus femoris MT and CSA were significantly correlated with predicted ASM (r = 0.428, *p* < 0.01; r = 0.462, *p* < 0.01). The area under the curve (AUC) for MT in identifying low muscle mass was 0.732 (95% CI 0.601 to 0.862, *p* = 0.001) at a cut-off value of 1.38 cm. CSA had an AUC of 0.789 (95% CI 0.678 to 0.901, *p* < 0.001) at a cut-off value of 4.48 cm^2^. CSA, MT, and PA were significant independent predictors of osteoporosis regardless of bone mineral density but not of FRAX parameters. **Conclusions**: Rectus femoris ultrasonography is a potentially reliable and rapid method for assessing muscle mass. Rectus femoris ultrasound parameters may serve as predictors of osteoporosis, independent of bone mineral density.

## 1. Introduction

Sarcopenia, or “muscle failure,” is a progressive and generalized condition primarily characterized by a decline in muscle strength associated with reduced muscle mass [1]. Its development is influenced by lifestyle factors as well as diminished anabolic signaling mediated by hormones such as growth hormone, IGF-1, and testosterone. Additionally, the aging process is accompanied by increased levels of pro-inflammatory cytokines, contributing further to sarcopenia. The phenomenon of “inflammaging” leads to a reduction in satellite cells, particularly those associated with type II muscle fibers, and increased intramuscular adipose tissue infiltration [2,3].

During menopause, the decline in estradiol has significant implications for both bone and muscle health. Estradiol is a critical regulator of the activation and proliferation of satellite cells—muscle stem cells responsible for growth and repair—linked to type II (fast-twitch) fibers and improves muscle metabolism by supporting mitochondria, protein synthesis, and muscle preservation [4,5]. Estradiol deficiency is associated with elevated pro-inflammatory cytokines, which in turn promote osteoclastogenesis and impair the proliferation and differentiation of activated satellite cells, reducing muscle regeneration and protein synthesis [6]. These cytokines also contribute to lipotoxicity, characterized by fatty infiltration in both bone marrow and skeletal muscle.

The coexistence of osteoporosis and sarcopenia was first described by Binkley et al. in 2009 [7]. This “hazardous duet” significantly increases the risk of fractures, frailty, and mortality, leading to substantial loss of independence in affected individuals [8,9]. In one study involving older adults with hip fractures, those with osteosarcopenia had a markedly higher mortality rate (15.1%) compared to individuals with osteoporosis alone (5.1%) [10].

While the diagnosis of osteoporosis is straightforward, the diagnosis of sarcopenia remains inconsistent due to variability in assessment methods and cut-off values proposed by different expert groups [11]. Muscle mass can be evaluated using dual-energy X-ray absorptiometry (DXA), bioimpedance analysis (BIA), computed tomography (CT), magnetic resonance imaging (MRI), and more recently, ultrasound. Although CT and MRI are considered gold standards, they are resource-intensive, time-consuming, and, in the case of CT, expose patients to radiation [12]. In response to these limitations, the SARCUS working group proposed the use and standardization of muscle ultrasound in 2018, as a low-cost and accessible alternative [13]. This investigative method can be used to evaluate both muscle mass (i.e., thickness and cross-sectional area) and architecture; the latter includes measuring the pennation angle, fascicle length, and echo intensity—features that reflect the force-generating capacity of muscle and the degree of adipose tissue infiltration [13,14].

The aim of our study was to correlate rectus femoris ultrasonography findings with a reference method (bioimpedance analysis) and to propose population-specific cut-off values, as well as to compare muscle ultrasound parameters between postmenopausal women with and without osteoporosis.

## 2. Materials and Methods

### 2.1. Study Population

A total of 88 postmenopausal women aged 50 to 85 years, presenting to the Endocrinology Department of Targu Mures County Hospital, Romania, were enrolled in the study between 1 January 2024 and 28 February 2025. All participants had undergone a DXA scan within the preceding 12 months using the Lunar Prodigy Primo DXA scanner (GE Healthcare, Madison, WI, USA). Bone mineral density was measured at the lumbar spine (L1–L4) and at the proximal femur (femoral neck and total hip). The scan mode was determined by the DXA operator according to standard clinical practice and the manufacturer’s recommendations for body size.

The osteoporosis cohort included women aged 50 years or older with diagnosed osteoporosis, defined by a DXA T-score ≤ −2.5, or osteopenia (T-score between −1.0 and −2.5) with a history of at least one fragility fracture. Participants were included in the control group if they were 50 years or older, had a T-score ≥ −1.0, and had no history of fragility fractures or signs of lumbar fractures on X-ray.

Exclusion criteria for both cohorts included hyperthyroidism, hyperparathyroidism, hypercortisolism, acromegaly, premature menopause, rheumatoid arthritis, systemic lupus erythematosus, ankylosing spondylitis, hematological malignancies, inflammatory bowel disease, celiac disease, liver cirrhosis, chronic kidney disease with a GFR ≤ 60 mL/min/1.73 m^2^, and neuromuscular or neurodegenerative disorders. Participants receiving antiepileptic treatment or current or past glucocorticoid therapy were also excluded.

All eligible participants who met the inclusion criteria during the designated study period were included in the study.

Data was collected to calculate the FRAX Plus score, including the following:

- Age
- Height and weight
- History of parental hip fracture
- Personal fracture history
- Smoking status
- Corticosteroid use
- Presence of rheumatoid arthritis
- Presence of secondary osteoporosis
- Alcohol consumption

### 2.2. Measurement of Muscle Strength

Handgrip strength was measured using a Camry digital dynamometer (CAMRY EH101, Sensun Weighing Apparatus Group Ltd., Zhongshan, China) following the standardized protocol recommended by the American Society of Hand Therapists (ASHT) [15]. Participants were seated with their shoulders adducted and neutrally rotated, elbows flexed at 90°, forearms in a neutral position, and wrists maintained in 0° to 30° extension and 0° to 15° ulnar deviation. Each participant performed three maximal grip efforts with a 1 min rest time between trials, and the highest value was recorded for analysis. Only the handgrip strength of the dominant side was performed.

### 2.3. Evaluation of Body Composition and Muscle Mass

#### 2.3.1. Bioimpedance Analysis

Body composition was assessed using the Tanita MC-780A Multi Frequency Segmental Body Composition Analyzer (Tanita Corporation, Tokyo, Japan). Measurements were performed in accordance with the manufacturer’s guidelines. Participants stood barefoot on the analyzer’s footpads and held the hand electrodes with arms extended slightly away from the body. The device uses multi-frequency bioelectrical impedance analysis (5 kHz, 50 kHz, and 250 kHz) to estimate segmental and whole-body parameters, including skeletal muscle mass. To estimate appendicular skeletal muscle mass (ASM), the equation by Sergei et al. was used [16]:

ASM (kg) = −3.964 + (0.227 × RI) + (0.095 × weight) + (1.384 × sex) + (0.064 × Xc).

(RI = resistance index = height in cm^2^/resistance; Xc = reactance)

Resistance and reactance were calculated using the trigonometric equations from impedance and the phase angle provided by the bioimpedance output.

#### 2.3.2. Rectus Femoris Ultrasonography

Muscle ultrasound was performed using the Esaote MyLab50 X-Vision system (Esaote S.p.A., Genoa, Italy) in B-mode, with a 12 MHz linear transducer, following the recommendations of the SARCUS working group [13,14]. Assessments were conducted on the dominant-side rectus femoris. Participants were positioned supine, with a 5 min rest period prior to the examination.

Measurements were taken at the midpoint between the greater trochanter and the superior border of the patella on the dominant leg.

**Figure 1:**
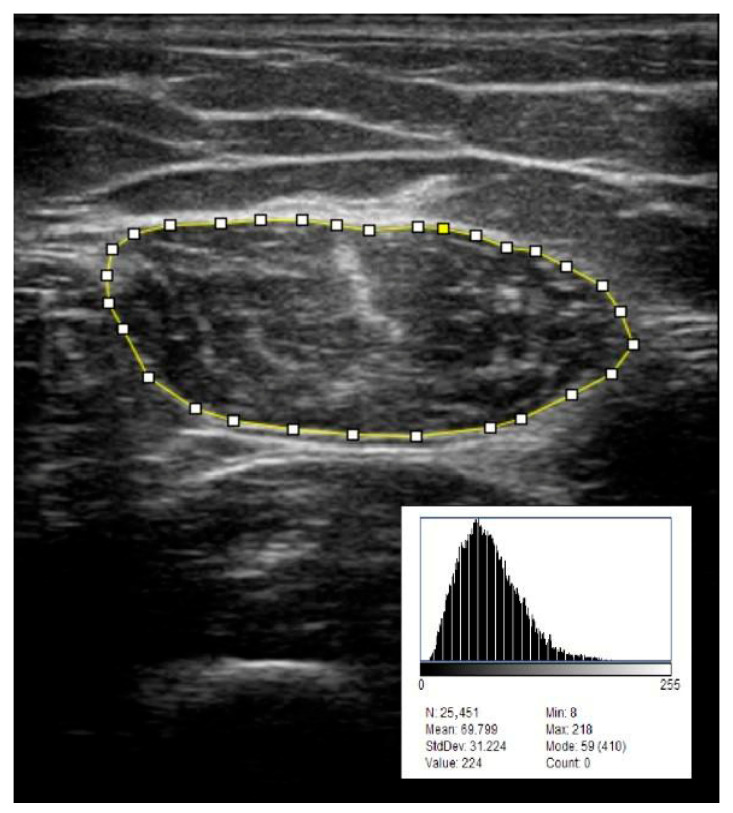
Assessment of echo intensity. The region of interest (ROI) outlined in yellow on the ultrasound image was analyzed for grayscale intensity. The histogram shows pixel values (0–255) and provides descriptive statistics including the number of pixels (N), mean, standard deviation (SD), minimum, maximum, and mode.

- Muscle thickness (MT) was measured in the transverse plane, from the superficial to the deep aponeurosis, at the center of the muscle.
- Cross-sectional area (CSA) was assessed in the transverse plane by tracing the muscle border as close as possible to the muscle fascia, without including it.
- Pennation angle (PA) was measured in the longitudinal plane, defined as the angle between a visible muscle fascicle and the deep aponeurosis, taken at the midportion of the muscle.
- Echo intensity (EI) was evaluated from transverse images captured at a fixed gain setting of 55%, using the ImageJ software (version 1.54g; National Institute of Health, Bethesda, MD, USA) as illustrated in Figure 1. Echo intensity values were expressed in grayscale units (0–255 scale). Echo intensity was evaluated in a subset of patients (*n* = 56).

All measurements were performed three times, and the average was used for analysis.

The study was conducted in accordance with the Declaration of Helsinki, and the protocol was approved by the Ethics Committee of the Targu Mures County Hospital (Project identification number: 12217, 1 November 2022).

#### 2.3.3. Statistical Analysis

Descriptive statistics were calculated for all variables. Normality was assessed using the Shapiro–Wilk test. Variables with a normal distribution are reported as mean ± standard deviation (SD), while non-Gaussian distributed variables are expressed as median and interquartile range (IQR).

For group comparisons, the independent samples *t*-test was used for normally distributed variables. Levene’s test was used to check the homogeneity of data variance. The Mann–Whitney U test was used for non-parametric variables. To adjust for potential confounding factors, analysis of covariance (ANCOVA) was performed. Assumptions of ANCOVA—including homogeneity of regression slopes, linearity, and normality of residuals—were tested prior to analysis.

Correlation analysis was conducted to examine the relationships between muscle ultrasound parameters, bioimpedance measurements, and functional performance measures. The Pearson’s correlation coefficient was used after assessing the normality and linearity of the data. Correlation strength was interpreted using the following thresholds:

- Weak: |r| = 0.10–0.29
- Moderate: |r| = 0.30–0.49
- Strong: |r| = 0.50–0.69
- Very strong: |r| ≥ 0.70

Receiver operating characteristic (ROC) curve analysis was performed for muscle thickness, cross-sectional area, pennation angle, and echo intensity to evaluate their ability to detect low muscle mass, defined as estimated ASM < 5.5 kg/m^2^ according to the criteria of the European Working Group on Sarcopenia in Older People 2 (EWGSOP2) [1]. The area under the curve (AUC), optimal cut-off values, sensitivity, and specificity were reported.

Binary logistic regression was conducted with “low muscle mass” and “osteoporosis” as dependent variables. The Box–Tidwell test was used to confirm the assumption of linearity between continuous predictors and the logit of the dependent variable. Multicollinearity was assessed using variance inflation factors (VIFs). Due to multicollinearity between lumbar and femoral bone mineral density (BMD), only lumbar BMD was included in the regression model, as a greater proportion of participants had osteoporosis at this site. Due to multicollinearity between muscle ultrasound parameters, each parameter was used in a separate logistic regression model adjusted for age, BMI, and BMD.

All statistical analyses were performed using IBM SPSS Statistics version 30 (IBM Corp., Armonk, NY, USA). *p* values < 0.05 were considered statistically significant.

## 3. Results

### 3.1. Patient Characteristics

A total of 88 postmenopausal women with a mean age of 65.7 ± 7.5 years were enrolled in the study. Osteoporosis was present in 59% of the participants. Low muscle strength (indicative of probable sarcopenia) was observed in 22% of the study population, while 24% of women exhibited low muscle mass. Sarcopenia, defined by the coexistence of both low muscle strength and low muscle mass, was diagnosed in 7% of the participants. Notably, all women with sarcopenia also had osteoporosis. Descriptive statistics and group comparisons, both unadjusted and adjusted for age and BMI, are presented in Table 1.

Women with osteoporosis were significantly older and had lower BMIs as compared to the control group. Muscle thickness was significantly lower in the osteoporosis group after adjusting for age and BMI, while the difference in cross-sectional area and echo intensity became non-significant after adjustment for BMI. Within the osteoporosis group, women with osteosarcopenia (*n* = 6) showed a downward trend in muscle thickness (1.3 cm ± 0.16), cross-sectional area (4.04 ± 0.45 cm^2^), and pennation angle (9.8° ± 1.4), along with higher echo intensity (114.8 ± 15). However, statistical comparison for this subgroup was limited by the small sample size.

Patient comorbidities and osteoporosis treatments are presented in Table 2.

### 3.2. Correlations Between Rectus Femoris Ultrasound Parameters, Bioimpedance, and Functional Parameters

Correlations between muscle ultrasound parameters and bioimpedance parameters as well as functional outcomes are displayed in Table 3. Ultrasound-derived muscle thickness and cross-sectional area showed moderate to strong correlations with appendicular skeletal muscle mass and sarcopenic index (SI). CSA also demonstrated moderate inverse correlations with EI.

### 3.3. Diagnostic Utility of Rectus Femoris Ultrasonography in Detecting Low Muscle Mass and Proposed Cut-Off Values

**Figure 2:**
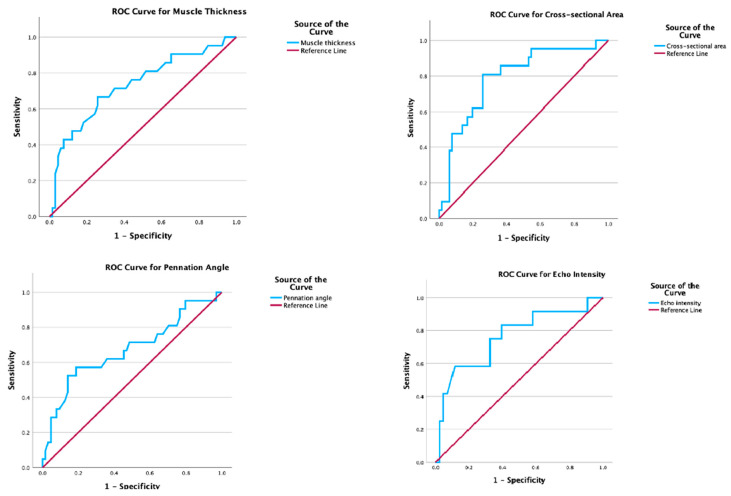
ROC curves for rectus femoris ultrasound parameters.

ROC curve analysis was performed to determine cut-off values for detecting low muscle mass using rectus femoris muscle ultrasound parameters which are displayed in Figure 2. The AUC for EI was 0.760 (SE = 0.085, 95% CI: 0.594–0.926, *p* = 0.006) with a sensitivity of 91.7% and specificity of 41.9% at a cut-off value of 71.125 greyscale units. PA had an AUC of 0.674 (SE = 0.074, 95%CI: 0.529–0.819, *p* = 0.017). The optimal cut-off value was 9.685°, resulting in a sensitivity of 52.4% and a specificity of 85.9%. The AUC for MT was 0.732 (SE = 0.067, 95%CI: 0.601–0.862, *p* = 0.001). Based on the Youden’s index the optimal cut-off point was 1.385 cm with a sensitivity of 66.7% and a specificity of 74%. CSA showed stronger diagnostic accuracy for detecting low muscle mass, with an AUC of 0.789 (SE = 0.057, 95%CI: 0.678 to 0.901, *p* < 0.001). The optimal cut-off value was 4.48 cm^2^ with a sensitivity of 81% and specificity of 74.2%.

Cross-sectional area proved to be the most accurate in detecting low muscle mass in ROC analysis. A binary logistic regression model was used to examine predictors of low muscle mass. After adjusting for age, height, and body fat percentage, CSA remained a significant independent predictor (OR = 0.33, 95% CI: 0.16–0.67, *p* = 0.002), indicating that larger CSA values were associated with significantly lower odds of having low muscle mass. Fat percentage was also a significant predictor (OR = 0.87, *p* = 0.008).

### 3.4. Rectus Femoris Ultrasound Parameters as Predictors of Osteoporosis

Logistic regression analysis was conducted to evaluate predictors of osteoporosis status. Rectus femoris CSA, MT, and pennation angle were identified as significant predictors of osteoporosis, independent of age, BMI, and BMD. In a model including only age, BMI, and BMD, the AUC was 0.860 (95% CI: 0.782–0.939, *p* < 0.001). Adding CSA to this model improved the AUC to 0.884 (95% CI: 0.813–0.954, *p* < 0.001). The individual logistic regression models are displayed in Table 4.

When FRAX parameters were incorporated into the models, the cross-sectional area, muscle thickness, and pennation angle were no longer significant predictors.

## 4. Discussion

### 4.1. Rectus Femoris Ultrasound Differences Based on Osteoporosis Status

The pathophysiology underlying muscular changes in postmenopausal women may involve hormonal, nutritional, and inflammatory mechanisms. Estrogen deficiency, vitamin D deficiency, and inadequate protein intake can negatively impact both muscle and bone. The interplay of osteokines, myokines, and adipokines regulates fat infiltration, muscle size, and bone metabolism through effects on inflammatory signaling, anabolic–catabolic balance, and mesenchymal stem cell differentiation [17,18]. The end-results of these processes were well illustrated by Nilwik et al. and Terracciano et al., who reported that age-related muscle loss is largely due to reductions in type II fiber size [19], and that type II fiber atrophy is predominantly found in women with osteoporosis, respectively [20].

We found that women with osteoporosis had a significantly decreased rectus femoris muscle thickness, even after adjusting for age and BMI, when compared to controls. However, the cross-sectional area did not follow this pattern.

Our result was only partially mirrored in a study by Yekta et al., that reported significant differences in gastrocnemius muscle thickness and cross-sectional area between women with osteosarcopenia and healthy controls, while highlighting the fact that this was not the case between the osteoporosis and control groups. It should be noted, however, that they found increased echo intensities in both the osteosarcopenia and osteoporosis groups [21]. Luo et al. observed reduced muscle size and increased echo intensity in the abdominal muscles of postmenopausal women with osteoporosis, though the inclusion of premenopausal controls in their study limits a direct comparison [22].

In our cohort, echo intensity differed between groups after adjusting for age, but the statistical significance was attenuated after adjusting for BMI. While the evaluation of echo intensity is useful in identifying muscular adipose tissue infiltration, BMI must be taken into consideration, as greater subcutaneous adiposity could potentially attenuate ultrasound signals. This phenomenon was previously reported by Müller et al., who stated that increased subcutaneous adipose tissue ultimately leads to an underestimation of echo intensity [23].

Although group differences in muscle function were not statistically significant in our study, the observed differences in muscle size suggest that muscle ultrasound could serve as an early marker of musculoskeletal deterioration—potentially identifying individuals at risk before functional impairment becomes evident.

### 4.2. Associations Between Rectus Femoris Ultrasonography and BIA Parameters

When comparing rectus femoris ultrasound measurements with bioimpedance-derived estimates of muscle mass, statistically significant correlations of moderate strength were found between muscle thickness and cross-sectional area with predicted appendicular skeletal muscle mass and sarcopenic index by BIA.

Furthermore, echo intensity demonstrated significant negative correlations with cross-sectional area, as well as with BIA-derived muscle mass and SI. This suggests that higher intramuscular fat infiltration, reflected by increased EI, is associated with lower muscle mass. Our findings are consistent with those of Yoshiko et al., who reported that individuals with higher EI had significantly lower skeletal muscle mass and SI, although no significant differences in strength were observed [24]. Akima et al. found that EI and muscle thickness of the rectus femoris were associated regardless of age [25].

In our population, functional parameters—specifically handgrip strength and gait speed—did not significantly correlate with EI. This contrasts with Fukumoto et al., who found a significant association between EI and maximal isometric knee extension strength [26]. Given that muscle loss typically begins in the lower extremities, elevated EI may serve as an early marker of strength loss not yet detectable through handgrip strength.

Pennation angle was strongly and positively correlated with muscle thickness and cross-sectional area; however, it did not significantly correlate with functional parameters.

Similarly, Dos Santos et al. also reported a significant correlation between pennation angle and muscle thickness, and none between handgrip strength and pennation angle in their study population [27].

### 4.3. Rectus Femoris MT and CSA Cut-Off Values for Detecting Low Muscle Mass

Rectus femoris muscle thickness demonstrated acceptable discriminative ability in identifying low muscle mass with an AUC of 0.732. The optimal cut-off value was 1.38 cm, yielding a sensitivity of 66.7% and specificity of 74%. Cross-sectional area outperformed muscle thickness, with an AUC of 0.789, a sensitivity of 81%, and a specificity of 74.2% at a cut-off value of 4.48 cm^2^.

Fukumoto et al. reported a sensitivity of 60% and specificity of 67% for muscle thickness in older women, using a cut-off of 1.43 cm, though the study did not specify whether the dominant or non-dominant leg was assessed [28]. Esme et al. found an AUC of 0.728 with 76% sensitivity and 69% specificity for detecting sarcopenia [29]. In our study, the low number of sarcopenic participants (*n* = 6) limited defining cut-off values for sarcopenia, therefore, low muscle mass was used.

Despite the encouraging potential of ultrasound to detect low muscle mass, significant heterogeneity exists across studies in terms of patient positioning, measurement sites, and the use of the dominant versus the non-dominant limb [30]. These differences lead to variability in cut-off values, highlighting the need for standardization to make this a valid diagnostic method that yields results with actionable, real-world application. While Perkisas et al. have proposed detailed recommendations and anatomical landmarks for standardizing measurement protocols, further studies are needed that actively implement these recommendations in diverse populations to establish validated, population-specific cut-off points [14].

### 4.4. Rectus Femoris Ultrasound Parameters as Predictors of Osteoporosis

In our logistic regression models, rectus femoris CSA, MT, and pennation angle measured via ultrasound emerged as independent predictors of osteoporosis after adjusting for age, BMI, and BMD. However, the inclusion of FRAX parameters—particularly fracture history—attenuated the associations, rendering them non-significant. This attenuation raises important considerations. It is possible that the predictive capacity of muscle ultrasound parameters overlaps with that of clinical fracture history. For instance, individuals with a history of fractures may experience reduced mobility or physical activity, leading to muscle atrophy and lower CSA, MT, and pennation angle. Alternatively, the relatively small sample size may have limited the statistical power to detect independent effects once FRAX variables were included.

Encouragingly, comparable findings have been reported using other imaging modalities. Park et al. demonstrated that CSA at the L3 vertebral level, assessed by CT, was an independent predictor of osteoporosis in hemodialysis patients [31]. Harvey et al. used high-resolution peripheral quantitative CT to measure both CSA and muscle density of the calf muscles in older men and found that both parameters were associated with incident hip fractures [32]. Notably, muscle density remained a significant predictor even after adjusting for BMD and FRAX, suggesting that muscle architectural metrics may offer fracture risk information that traditional tools might miss.

Although this study did not include fracture outcomes, the potential role of rectus femoris ultrasound parameters in identifying individuals at higher risk of osteoporosis—independent of BMD—merits further investigation. Future studies with larger sample sizes and prospective follow-up could clarify whether muscle ultrasound can enhance clinical risk stratification alongside tools like FRAX. Moreover, future studies involving multiple operators performing ultrasonography would enhance the methodology and are a crucial next step in ensuring the validity of our results.

### 4.5. Limitations

This study has several limitations. First, it was a single-center, cross-sectional study with a relatively small sample size, which limits the generalizability of the findings. Moreover, sample size calculation was not performed prior to enrollment.

Additionally, we used bioelectrical impedance analysis as the reference method to estimate muscle mass. As an indirect technique, BIA is influenced by factors such as hydration status and may overestimate muscle mass in individuals with higher BMI. We applied the predictive equation proposed by Sergei et al. to estimate appendicular skeletal muscle mass. Although this equation is validated, it was not developed in our population, and the bioimpedance device used in our study differs from that used in the original validation study, potentially leading to an underestimation of ASM.

There was also a statistically significant difference in age and BMI between the osteoporosis and control groups; these variables were adjusted for in the group comparison, although the possibility of residual confounding cannot be excluded.

Moreover, muscle ultrasound was performed by a single operator, which may introduce measurement bias and prevent assessment of inter-rater reliability. Future studies should involve multiple operators to evaluate inter-rater reliability and confirm the consistency and robustness of the technique.

Lastly, while we excluded diagnosed conditions that affect both bone and muscle, undiagnosed diseases and differences in lifestyles may still have influenced our results.

## 5. Conclusions

In our study rectus femoris ultrasound parameters correlated significantly with estimated ASM, with acceptable sensitivity and specificity in identifying low muscle mass, in particular cross-sectional area. Measurements of muscle thickness and cross-sectional area may provide a quick and practical method to assess muscle quantity. Rectus femoris cross-sectional area, muscle thickness, and pennation angle were significant predictors of the presence of osteoporosis independent of bone mineral density, but not FRAX.

## Figures and Tables

**Table 1:** Patient characteristics and comparison between groups.

Parameter	Control Group (Mean ± SD or Median [IQR])	Osteoporosis Group (Mean ± SD or Median [IQR])	*p*-Value (Unadjusted)	*p*-Value (Age and BMI Adjusted)
Age (years)	63.7 ± 6.9	67.0 ± 7.9	0.040	—
BMI (kg/m^2^)	29.24 ± 4.56	26.69 ± 4.67	0.013	—
Fat Percentage (%)	37.5 (7.0)	36.35 (10.1)	0.200	—
ASM/height^2^ (kg/m^2^)	6.29 ± 0.74	5.84 ± 0.67	0.004	0.143
Phase Angle (°)	5.3 ± 0.76	4.9 ± 0.77	0.010	0.139
Handgrip Strength (kg)	22.58 ± 5.8	21.19 ± 5.1	0.237	—
Gait Speed (m/s)	1.44 ± 0.38	1.37 ± 0.41	0.428	—
Muscle Thickness (cm)	1.54 ± 0.17	1.39 ± 0.19	<0.001	0.016
Cross-sectional area (cm^2^)	5.26 ± 0.95	4.63 ± 1.02	0.004	0.090
Pennation Angle (°)	11.6 ± 1.7	10.9 ± 1.68	0.075	—
Echo Intensity (g.u.)	73.88 ± 27.5	92.03 ± 25.0	0.013	0.1
BMD Femur (g/cm^2^)	0.880 ± 0.1	0.726 ± 0.95	<0.001	—
BMD L1–L4 (g/cm^2^)	1.115 ± 0.16	0.912 ± 0.14	<0.001	—

**Table 2:** Patient comorbidities and treatments.

Parameter	Control Group, *n* (%)	Osteoporosis Group, *n* (%)
Parental Fracture History	3 (8.3%)	6 (11.5%)
Smoking	5 (13.9%)	10 (19.2%)
Hypertension	21 (58.3%)	36 (69.2%)
Type 2 Diabetes	2 (5.6%)	6 (11.5%)
Obesity	13 (36.1%)	11 (21.2%)
Treated Hypothyroidism	16 (44.4%)	18 (34.6%)
Vitamin D Supplementation	28 (77.8%)	48 (92.3%)
Osteoporosis Treatment		
Bisphosphonates		16 (30.8%)
Denosumab		9 (17.3%)
Teriparatide		19 (36.5%)
Treatment Holiday		5 (9.6%)
Newly Diagnosed, No Treatment		3 (5.8%)

**Table 3:** Correlation between ultrasound, bioimpedance, and functional parameters.

	MT	CSA	PA	EI	ASM	SI	PhA	HGS	GS
MT	1	0.794 **†	0.589 **†	−0.278 *	0.428 **†	0.393 **†	0.233 *	0.321 **	0.239 *
CSA	0.794 **†	1	0.717 **†	−0.490 **†	0.462 **†	0.513 **†	0.298 **	0.305 **	0.175
PA	0.589 **†	0.717 **†	1	−0.323 *	0.280 **	0.296 **	0.027	0.315 **	0.236 *
EI	−0.278 *	−0.490 **†	−0.323 *	1	−0.507 **†	−0.515 **†	−0.316 *	−0.164	0.154
ASM	0.428 **†	0.462 **†	0.280 **	−0.507 **†	1	0.839 **†	0.425 **†	0.214 *	−0.045
SI	0.393 **†	0.513 **†	0.296 **	−0.515 **†	0.839 **†	1	0.533 **†	0.063	−0.088
PhA	0.233 *	0.298 **	0.027	−0.316 *	0.425 **†	0.533 **†	1	0.193	0.107
HGS	0.321 **	0.305 **	0.315 **	−0.164	0.214 *	0.063	0.193	1	0.413 **†
GS	0.239 *	0.175	0.236 *	0.154	−0.045	−0.088	0.107	0.413 **†	1

*p* < 0.05 (*), *p* < 0.01 (**), *p* < 0.00139 Bonferroni-corrected significance (†). MT = Muscle Thickness, CSA = Cross-sectional Area, PA = Pennation Angle, EI = Echo Intensity, ASM = Appendicular Skeletal Muscle Mass, SI = Sarcopenia Index, PhA = Phase Angle, HGS = Handgrip Strength, GS = Gait Speed.

**Table 4:** Logistic regression models of osteoporosis predictors.

Parameter *	OR (95% CI)	*p*-Value
Muscle Thickness (cm)	0.025 (0.001–0.773)	0.035
Cross-sectional Area (cm^2^)	0.466 (0.242–0.897)	0.022
Pennation Angle (°)	0.644 (0.426–0.975)	0.037
Echo Intensity (g.u.)	1.034 (0.998–1.072)	0.065

* All predictors were tested in separate logistic regression models adjusted for age, BMI, and bone mineral density. A complete summary of the logistic regression analysis, showing all covariates included in the models, can be found in Supplementary Table S1.

## Data Availability

The raw data supporting the conclusions of this article will be made available by the authors on request.

## Supplementary Materials

The following supporting information can be downloaded at: https://www.mdpi.com/article/10.3390/jcm14186531/s1.

## Abbreviations

The following abbreviations are used in this manuscript:

DXA	Dual-energy X-ray absorptiometry
CT	Computed tomography
MRI	Magnetic resonance imaging
BIA	Bioimpedance analysis
MT	Muscle thickness
CSA	Cross-sectional area
PA	Pennation angle
EI	Echo-intensity
ASM	Appendicular skeletal muscle mass
SI	Sarcopenic index
PhA	Phase angle
HGS	Handgrip strength
GS	Gait speed
EWGSOP2	European Working Group on Sarcopenia in Older People 2
SARCUS	SARCopenia through UltraSound
FRAX	Fracture risk assessment tool
BMD	Bone mineral density
