# Supplementary material for: Bridging Muscle and Bone Health: Rectus Femoris Ultrasound Parameters Predict Osteoporosis and Identify Low Muscle Mass in Romanian Postmenopausal Women
Source: J Clin Med. 2025 Sep 17;14(18):6531. doi: 10.3390/jcm14186531 (PMC12470516; doi:10.3390/jcm14186531)
Supplement: Supplementary file 1 [file jcm-14-06531-s001.zip › jcm-3820305-supplementary.pdf]

Table S1

Model (Main Predictor)	Predictor	B	SE	OR	95% CI	p-value
**Model 1: Muscle Thickness**	BMD	-10.471	2.719	0.000	[0.000, 0.006]	<0.001
	Age	0.068	0.046	1.070	[0.977, 1.172]	0.143
	BMI	-0.082	0.069	0.921	[0.805, 1.054]	0.232
	MT	-3.682	1.747	0.025	[0.001, 0.773]	0.035
**Model 2: Cross-Sectional Area**	BMD	-11.651	2.877	0.000	[0.000, 0.002]	<0.001
	Age	0.080	0.047	1.083	[0.988, 1.187]	0.087
	BMI	-0.062	0.071	0.940	[0.817, 1.081]	0.386
	CSA	-0.764	0.334	0.466	[0.242, 0.897]	0.022
**Model 3: Pennation Angle**	BMD	-12.372	3.010	0.000	[0.000, 0.002]	<0.001
	Age	0.080	0.046	1.083	[0.990, 1.185]	0.081
	BMI	-0.100	0.069	0.905	[0.791, 1.035]	0.146
	PA	-0.439	0.211	0.644	[0.426, 0.975]	0.037
**Model 4: Echo Intensity**	BMD	-15.107	4.524	0.000	[0.000, 0.002]	0.001
	Age	0.177	0.076	1.193	[1.028, 1.385]	0.020
	BMI	-0.037	0.103	0.964	[0.787, 1.181]	0.723
	EI	0.034	0.018	1.034	[0.998, 1.072]	0.065
